# Chloride Balance in Preterm Infants during the First Week of Life

**DOI:** 10.1155/2012/931597

**Published:** 2012-03-08

**Authors:** Silvia Iacobelli, Elsa Kermorvant-Duchemin, Francesco Bonsante, Alexandre Lapillonne, Jean-Bernard Gouyon

**Affiliations:** ^1^Neonatology and NICU, GHSR, CHR, BP 350, 97448 Saint Pierre Cedex, Réunion, France; ^2^Centre d'Etudes Périnatales de l'Océan Indien, Centre d'Investigation Clinique et d'Epidémiologie Clinique (CIC-EC) CHR, 97410 Saint Pierre, Réunion, France; ^3^Department of Neonatology, AP-HP, Groupe Hospitalier Cochin-Saint-Vincent de Paul, 75014 Paris, France; ^4^Paris Descartes University, 75270 Paris, France; ^5^NICU, Department of Paediatrics, University of Dijon, 21034 Dijon Cedex, France

## Abstract

*Objective*. To describe the chloride balance in infants born 25–32-week gestation, analyze the association of chloride changes with hydroelectrolytic status and their relationship with perinatal conditions, morbidities, and neurological outcome. *Methods*. For 7 days after birth, sodium and chloride balance, plasma potassium, phosphate, and total carbon dioxide (tCO_2_) were prospectively determined and strong ion difference (SID) calculated. Three multivariate regression analyses were performed to identify factors associated with high plasma chloride concentration, low SID, and low tCO_2_. *Results*. 107 infants were studied. Plasma chloride concentration was significantly positively associated with plasma sodium concentration. Higher plasma chloride and lower SID were significantly associated with lower plasma tCO_2_. Chloride intake was the main independent factor associated with high plasma chloride, low SID, and low plasma tCO_2_, with lesser contribution of sodium intake and low gestational age (GA). Also, patent ductus arteriosus and birth weight loss were independent factors affecting plasma chloride and SID. Neither high chloride levels nor low SID were associated to impaired neurological outcome. *Conclusions*. In preterm infants, chloride balance is influenced by GA and by interrelationship between sodium and chloride intake. High chloride levels are associated with metabolic acidosis but not related to increased risk of impaired neurological outcome.

## 1. Introduction

Transition to the extrauterine environment is associated with major changes of body water and salt composition in the premature baby [1]. Changes in plasma sodium concentrations have been particularly studied, and large variations in sodium levels have shown a relationship with impaired outcome [2]. On the contrary, metabolism of chloride (Cl^−^), the major anion of the extracellular fluids, has been rarely investigated. Even if chloride balance usually parallels that of sodium, and so it is strictly correlated to the extracellular volume balance, chloride losses and excretion can also occur independently from sodium, mainly in equilibrium with bicarbonate status. Therefore, hyperchloraemia is often associated with metabolic acidosis, a causative factor for intraventricular haemorrhage and other morbidities in preterm babies [3]. The concept of “strong ion difference” (SID) is used to help explain “metabolic” acid base abnormalities associated with changes in chloride concentration [4]. According to the “Stewart's approach,” three independent variables determine pH in plasma, by changing the degree of water dissociation into hydrogen ions. These three variables are the SID, the partial pressure of carbon dioxide CO_2_ (pCO_2_), and the total concentration of week acids (primary albumin). A decrease in the SID will result in an acidifying effect on plasma. The SID is calculated as the charge difference between the sum of measured strong cations (Na^+^, K^+^, Ca^2+^, and Mg^2+^) and measured strong anions (Cl^−^, lactate) [5]. A strong ion is defined as one that is almost completely dissociated at physiological pH. As both Na^+^ and Cl^−^ are the major strong ions in plasma, the SID calculated as the simply difference between sodium and chloride represents one independent variable determining the hydrogen ion and the bicarbonate ion concentrations; an increase in the plasma Cl^−^ relative to Na^+^ decreases the plasma SID and lowers the pH [6].

To our knowledge, no study has explored the chloride balance during the first week of life or the impact of changes in plasma chloride levels on perinatal morbidity and neurological outcome in preterm infants. The present investigation was carried out to enlighten these two points.

## 2. Patients and Methods

### 2.1. Study Population

From January 2007 to May 2008 all consecutive infants born below 33 weeks of gestational age (GA) and admitted to the neonatal intensive care unit (NICU) of Dijon University Hospital within 6 hours after birth were eligible. Noninclusion criteria were major congenital anomalies. A criterion for secondary exclusion was death within the first week.

The research protocol was authorized by the Ethics Committee of the Hospital. Informed, signed parental consent was obtained.

Parenteral nutrition (PN) was administered by eight different PN bags commercially batch produced with increasing nutrient intake for day 1 to day 7 of life in infants with central venous line [7]. Infants without central venous line also received PN by commercially batch-produced bags at lower osmolarity and nutrient intake. 10% dextrose or sterile water for injection at choice could be added to PN bags by retrograde continuous infusion in order to achieve the required water supply. Guidelines for daily prescription of water were provided. These guidelines recommended starting fluid intake at 80 mL/kg/day and then giving fluid input to allow a daily weight loss from 2 to 4% and to maintain plasma sodium and potassium concentrations within 135 to 145 mmol/L and 4–6 mmol/L, respectively [8]. Starting sodium, potassium- and chloride intake was at day 3.

In the purpose to assess short- and middle-term neurological outcome in this population, cerebral ultrasounds were realized during the infant hospital stay and neurological examination at 18 months of corrected age.

Cranial ultrasounds were performed by experienced examiners (neonatologists or radiologists) according to the following protocol: day 1, 3, 7, 10, 15, and then at least every 2 weeks or more often as clinically indicated, until discharge.

Severe abnormal cerebral ultrasound was defined as severe (grade 3 or 4) intraventricular hemorrhage (IVH) and/or cystic periventricular leukomalacia (c-PVL) occurring before infants discharge from hospital. IVH was graded at cerebral ultrasound according to Papile et al. [9]. c-PVL was defined by cranial ultrasound as an area of increased echogenicity of the periventricular white matter in acute phase which subsequently evolved into cystic lesion [10].

Children were subjected to a detailed physical and neurological examination at 18 months of corrected age, in order to assess tone, reflexes, posture, and movements. We used the definition of cerebral palsy proposed by the European Cerebral Palsy Network [11].

### 2.2. Data Collection

For the 7 days after birth, plasma sodium, potassium, chloride, phosphate, and total carbon dioxide (tCO_2_) were determined daily. We daily calculated the plasma SID as the difference between sodium and chloride. Base excess (BE), bicarbonate (HCO_3_^−^), pCO_2_, and pH were performed according to clinician decision.

Day 1 data were obtained on a blood sample taken at 12 hours of life. Day 2 blood sample was taken 24 hours later.

Sodium, potassium, and chloride intake from intravenous, oral fluids and drugs administration during the study period was recorded from the infant chart. Intravenous flushes and sodium and chloride administration by drugs, or transfusions were taken into account when calculating fluid and electrolyte intake.

Daily, consecutive 8-hour urine collection starting 4 hours before the blood sampling was performed by using a plastic bag. Urine was analyzed for sodium and chloride concentrations. Plasma and urine sodium and chloride, plasma potassium, phosphate, and tCO_2_ concentrations were measured by an Ortho Clinical Diagnostic analyzer (Rochester, USA), which uses direct potentiometry. Blood gas was analyzed on the Radiometer ABL 700 blood gas analyzer.

Chloride and sodium balance were defined, respectively, as the difference between chloride intake and urinary excretion and sodium intake and urinary excretion was expressed as mmol/kg/day.

### 2.3. Statistics

Data from categorical variables were analyzed using *χ* ^2^ test or Fisher exact test for small samples. Continuous variables were expressed as mean ± standard deviation, and differences between groups were analysed using ANOVA or Kruskal-Wallis analysis of variance for not normally distributed data. Statistical tests were performed with SAS software 8.2. Results were considered statistically significant at a 5% level.

We measured the relationship between both plasma chloride concentration and plasma SID with plasma sodium, phosphate, tCO2, pH, BE, and HCO_3_^−^.

Furthermore, we realized for each of the following parameters (plasma chloride concentration, plasma SID, and tCO_2_) a univariate analysis of variance in order to explore their association with water, energy, amino acids, phosphate, sodium, potassium, and chloride intakes and also their association with all the perinatal variables summarized in Table 1. For each of the above parameters, variables significant at a *P* level <0.20 at the univariate analysis were entered into a backward selection analysis of variance.

## 3. Results and Discussion

### 3.1. Results

During the study period, 147 neonates born below 33 weeks of gestation were hospitalized in our NICU. Of these, 129 were admitted within the 6th hour of life. Among them, 12 were not enrolled in the study: 1 due to major congenital anomalies, 9 due to omission of the attending physician, and 2 due to refused parental consent. So, 117 infants were entered into the study. Among them, 10 were excluded secondary: 2 because they died during the first week, 2 as they were transferred to other units, and 6 due to difficulties of blood sampling. Finally, 107 infants were entered into the study. Table 1 shows antenatal and postnatal characteristics of the study population.

Chloride concentration values were available for 95, 96, 98, 98, 91, 92, and 88% of infants, and sodium concentration values were available for 98, 100, 99, 99, 95, 95, and 92% from day 1 to 7. tCO_2_ values were available for 87, 83, 86, 87, 83, 83, and 77% of infants from day 1 to 7. Arterial blood gas was performed in 37% of the blood samples; when these data were available, the analysis showed that plasma chloride and SID were significantly associated with tCO_2_, BE, and HCO_3_^−^ and that tCO_2_ was the main factor independently associated with higher plasma chloride and lower SID. Therefore, tCO_2_ was the biochemical parameter used to express the results concerning the relationship of chloride with metabolic acid base balance.

**Figure 1:**
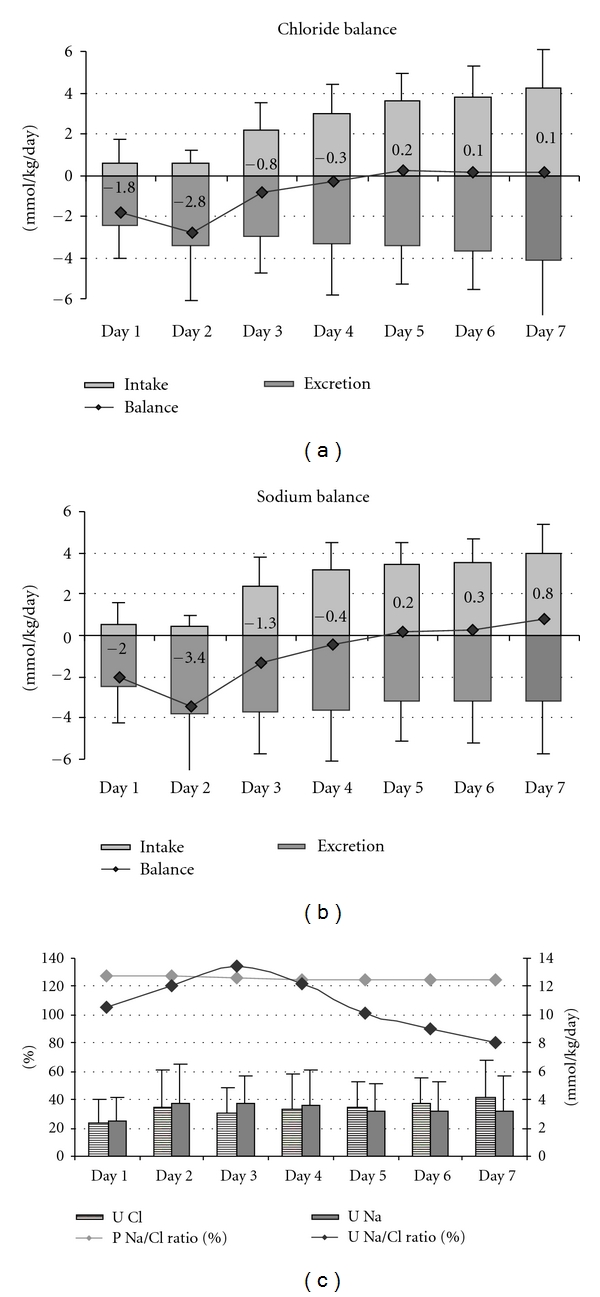
Chloride and sodium intake and balance during the first week of life in 107 infants <33 weeks of GA hospitalized in NICU.

**Figure 2:**
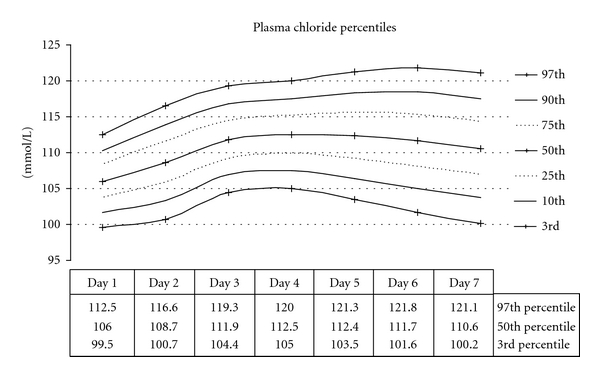
Plasma chloride percentiles during the first week of life in 107 infants <33 weeks of GA hospitalized in NICU (*P* value = 0.000001 at Kruskal-Wallis one-way analysis of variance).

Figure 1 shows the chloride and sodium intake, urinary excretion and balance during the study period.  Figure 2 shows plasma chloride percentiles during the first week of life in the study population. The mean plasma chloride value during the study period was 110.5 ± 5.0 mmol/L. Chloride concentration was equal or above 120 mmol/L in 4% of plasma samples and in 14% of patients. Hypernatraemia defined as plasma sodium >150 mmol/L occurred in 1.9% of infants.

Plasma chloride and sodium profiles were similar, while urinary chloride concentration did not parallel urinary sodium excretion (Figure 1).

**Figure 3:**
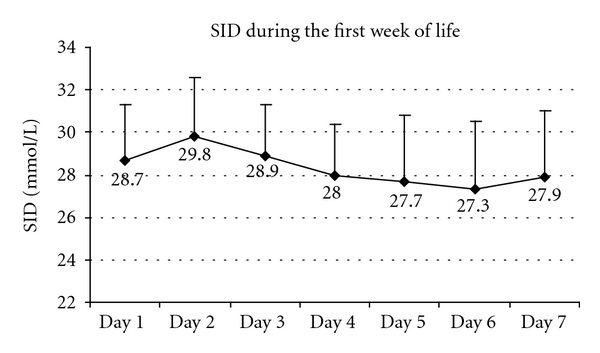
Strong ion difference (SID) during the first week of life in 107 infants <33 weeks of GA hospitalized in NICU (*P* value = 0.000001 at Kruskal-Wallis one way analysis of variance).

Figure 3 shows the evolution of the plasma SID during the first week of life.

Plasma chloride concentration was strongly positively associated with plasma sodium concentration (*r* ^2^ = 0.66; *P* < 0.000001); a negative significant association was described with plasma tCO_2_ (*r* ^2^ = 0.13; *P* < 0.01) and phosphate (*r* ^2^ = 0.05; *P* < 0.05). Plasma SID was significantly positively associated with plasma tCO_2_ (*r* ^2^ = 0.28; *P* < 0.001) and phosphate (*r* ^2^ = 0.11; *P* < 0.01).

Clinical factors associated with plasma chloride concentration at the univariate analysis were weight loss % of birth weight (BW), GA, acute anaemia at birth, respiratory distress syndrome (RDS) requiring surfactant, hypotension requiring treatment, hemodynamically significant patent ductus arteriosus (hsPDA), acute renal failure, intraventricular haemorrhage (IVH) grade 3-4, necrotizing enterocolitis (stage 2 or more of the Bell classification), chloride, sodium, phosphate, amino acid, and water intakes and day of life (data not shown). Among them, chloride intake, hsPDA, weight loss % of BW, sodium intake, and GA remained independent factors associated with plasma chloride at the multivariate analysis (Table 2). Factors associated with plasma SID at the univariate analysis were GA, acute anaemia at birth, RDS-requiring surfactant, hypotension requiring treatment, IVH grade 3-4, hsPDA, chloride, sodium, phosphate, and amino acid intake, and day of life (data not shown). Among them, chloride intake, hsPDA, sodium intake, phosphate intake and GA remained independent factors associated with plasma SID at the multivariate analysis (Table 2).

Factors associated with lower plasma tCO_2_ at the univariate analysis were GA, RDS requiring surfactant, hypotension requiring treatment, hsPDA, low Apgar score at 1 minute of life, sodium, chloride, phosphate and amino acid intake and day of life (data not shown). Among them, chloride and sodium intakes and GA remained independent factors associated with plasma tCO_2_ at the multivariate analysis (Table 2).

### 3.2. Discussion

To our knowledge this is the first prospective study showing the chloride balance in very preterm infants during the first week of life. Our data found a strong link between chloride and sodium metabolism. These results are consistent with others; Day et al. [13] showed that in very low birth weight infants (VLBWI) who developed hyponatraemia during the second and the third week of life, plasma chloride concentration paralleled plasma sodium, and this also occurred in sodium supplemented VLBWI between 2 and 6 weeks of age [14]. In the same infants chloride excretion in urine did not parallel that of sodium [14]. Our results also showed that urinary chloride excretion is independent from that of sodium since the first week of postnatal life. In our series weight loss % of BW was one of the clinical factors significantly associated to high chloride levels. This confirms that the strict correlation between sodium and chloride metabolism depends on parallel sodium and chloride changes due to the extracellular volume contraction after birth. 

Our study also disclosed a wide variability of chloride levels during the first week of life with mean plasma concentrations much higher when compared to the range usually considered as normal for preterm babies [15]. Higher chloride levels were detected in infants with lower GA. As reported by others [16], higher plasma chloride concentration and signs of metabolic acidosis (low SID and tCO_2_) were significantly associated with higher chloride intake in our study. Previous reports have already shown that a reduction in the chloride load by using acetate salts can be safely achieved and may even decrease plasma chloride levels and metabolic acidosis in preterm infants on PN during the first days of life [17, 18]. Groh-Wargo et al. underlined the effect of chloride from normal saline flushes in increasing the total chloride load in LBWI on PN [16]. Our results showed in addition that higher sodium and phosphate intake was associated to lower plasma chloride and lower metabolic acidosis in preterm infants on PN. The above suggests that sodium given in a salt formulation not containing chloride may be protective against metabolic acidosis and high chloride levels, and that the interrelationship between sodium and chloride intakes should be looked at as it can interfere with acid base homeostasis. Concerning the inverse association of phosphate intake and metabolic acidosis, we speculate that this could depend on the fact that dietary phosphate intake may affect renal regulation of plasma bicarbonate and so the renal defence against metabolic acidosis, as already proven in animal models and adults [19]. Of course this point needs to be investigated in future prospective studies. 

 Even if no previous studies have specifically explored the association of changes in plasma chloride levels and neonatal neurological morbidities, concerns have been expressed about the finding that blood chloride concentration had a significant linear correlation with metabolic acidosis, which is thought to be a causative factor in intraventricular haemorrhage in preterm [3]. Moreover, plasma chloride concentration has also a strong correlation with plasma sodium concentration, and several retrospective investigations focused on natraemia have reported that changes in sodium levels, as well neonatal hyper- or hyponatraemia, were associated to increased risk of severe cerebral haemorrhage and cerebral palsy [2, 20]. 

This cohort of very preterm infants provided important additional information, as in our study the only clinical variable associated with both high chloride levels and low SID was hsPDA, and this could be explained as infants with this pathological condition may exhibit metabolic acidosis [21]. Neither high chloride levels nor low SID were associated to impaired neurological outcome (severe abnormal cerebral ultrasound or cerebral palsy). It is worthy to note that the incidence of hypernatraemia was very low in our study, especially when compared to previous reports [22, 23]. 

Finally it is interesting to remind that chloride depletion too may represent a contributing cause of morbidity and mortality in preterm populations, as proven by human and animals studies [24, 25], but in our cohort low chloride levels were not correlated to increased morbidity. 

The low incidence of electrolyte disturbance-related morbidity in our population was probably due to careful management of fluid and electrolyte balance advocated by the unit guidelines and based on the strict monitoring of infants hydroelectrolytic status. 

## 4. Conclusion

This study allowed describing the postnatal chloride balance in preterm infants hospitalized in NICU. Plasma chloride levels during the first week are higher in infants born at lower GA, and they are correlated with plasma sodium levels and biological markers of metabolic acidosis. In this carefully monitored population, changes in plasma chloride levels were not associated to poor neurological outcome in the short and middle term. 

## Figures and Tables

**Table 1:** Characteristics of 107 infants <33 weeks of GA hospitalized in NICU.

Characteristics at birth	%
Male gender	54.2
BW	1316 ± 361^¤^
GA	30.0 ± 1.6^#^
SGA	25.4
Apgar score <3 at 1 minute of life	11.2

Prenatal characteristics	
Antenatal steroids	77.6
Caesarean section	79.4

Postnatal characteristics	
Central venous line	62.6
Acute anaemia at birth	13.2
Body weight loss >15% of BW	11.2
RDS-requiring surfactant	66.4
Early onset sepsis	6.5
Hypotension-requiring treatment	13.1
Acute renal failure*	11.2
HsPDA	36.4
Oxygen dependency beyond 36 wks PCA	12.3
Necrotizing enterocolitis	2.8
Severe abnormal cerebral ultrasound^†^	1.8
Death after the first week of life	0
Cerebral palsy at 18 months of PCA	2.1

(NICU) Neonatal Intensive Care Unit; (BW) birth weight; (GA) gestational age; (SGA) small for gestational age; (RDS) respiratory distress syndrome; (HsPDA) hemodynamically significant patent ductus arteriosus; (PCA) postconceptional age.

^¤^g (mean ± SD), ^#^weeks (mean ± SD), *according to [12], ^†^intraventricular haemorrhage grade 3 or 4 and/or periventricular leukomalacia.

**Table 2:** Factors associated with plasma chloride (mmol/L), SID, and tCO_2_ levels (mmol/L) at multivariate analysis in 107 infants <33 weeks of GA.

	*P* value	Incremental *r* ^2^	Beta coefficient
Plasma chloride*			
Chloride intake	<0.001	0.14	+
HsPDA	<0.01	0.03	+
Sodium intake	<0.01	0.02	−
Weight loss % of BW	<0.01	0.02	+
GA	<0.05	0.01	−

SID**			
Chloride intake	<0.001	0.09	−
HsPDA	<0.01	0.04	−
Sodium intake	<0.05	0.01	+
Phosphate intake	<0.05	0.01	+
GA	<0.05	0.01	+

tCO_2_***			
Chloride intake	<0.001	0.13	−
GA	<0.01	0.02	+
Sodium intake	<0.05	0.01	+

(SID) strong ion difference; (tCO_2_) total carbon dioxide; (hsPDA) hemodynamically significant patent ductus arteriosus; (BW) Birth weight; (GA) gestational age;

*General *r* ^2^ = 0.22; **General *r* ^2^ = 0.16  ***General *r* ^2^ = 0.16.
